# Validation of growth enhancing, immunostimulatory and disease resistance properties of *Achyranthes aspera* in *Labeo rohita* fry in pond conditions

**DOI:** 10.1016/j.heliyon.2019.e01246

**Published:** 2019-02-18

**Authors:** Neelesh Kumar, JaiGopal Sharma, Samar Pal Singh, Amarjeet Singh, V. Hari Krishna, Rina Chakrabarti

**Affiliations:** a Department of Biotechnology, Delhi Technological University, Bawana Road, Delhi 110 042, India; b Aqua Research Lab, Department of Zoology, University of Delhi, Delhi 110 007, India; c CIFE Rohtak Centre, Lahli, Rohtak, Haryana 124 411, India

**Keywords:** Immunology, Zoology

## Abstract

The immunostimulatory and disease resistance properties of herb *Achyranthes aspera* L. (Amaranthaceae) were evaluated in rohu *Labeo rohita* in pond. Rohu fry (1.9 ± 0.08 g) were cultured in hapas (25 hapa^−1^) set inside a pond and were fed with two experimental diets containing 0.5% seeds (D1) and leaves (D2) of *A. aspera* and control diet (D3). Fish were challenged with *Aeromonas hydrophila* after 80 days. The cumulative mortality rate of fish was significantly (*P* < 0.05) higher in D3 (28–48%) compared to others. Average weight was significantly (*P* < 0.05) higher in D1 (6.5–12.5%) compared to other treatments. Myeloperoxidase and nitric oxide synthase levels were significantly (*P* < 0.05) higher in D1 and D2 compared to D3. Thiobarbituric acid reactive substances and carbonyl protein levels were significantly (*P* < 0.05) lower in hepatopancreas and kidney of D1 compared to others. In hepatopancreas, the expressions of lysozyme C, loysozyme G, TNF-α, IL-10 and IL-1β were significantly (*P* < 0.05) higher in D1 compared to others. This treatment was followed by D2. In kidney, lysozyme G and TNF-α levels were significantly (*P* < 0.05) higher in D1 and D2 compared to D3. Whereas, IL-10 and IL-1β were significantly (*P* < 0.05) down-regulated and up-regulated, respectively in kidney of D2. There was up-regulation (*P* < 0.05) of TLR-4 in hepatopancreas and kidney of D1 and D2 diets fed rohu, respectively compared to others.

## 1 Introduction

The application of herbal immunostimulants has been increasing rapidly to control the disease in aquaculture [1]. Several plant ingredients show promising results viz. miers, *Tinospora cordifolia* leaf extract [2], mango, *Magnifera indica* kernel [3], *Solanum trilobatum* leaf [4], green tea [5], guava *Psidium guajava* leaves [6, 7], ginger *Zingiber officinale* [8]. All these studies are performed in the control laboratory conditions. Validation of these studies is required in the ponds, the most important source for aquaculture production in Asian countries. *Achyranthes aspera* L., a member of the Family Amaranthaceae is commonly found as weed in various parts of India. The bushy herb can be grown in the field regularly. Roots and seeds of *A. aspera* showed immunostimulatory and disease resistance properties in fishes in the laboratory conditions. The supplementation of *A. aspera* in diets stimulates specific and non-specific immune systems of *Catla catla* (catla), *Cyprinus carpio* (common carp) and *Labeo rohita* (rohu) [9, 10, 11, 12, 13]. The supplementation of seeds of *A. aspera* in diet also enhances the growth of fish. Even it protects the early larvae of carps from harmful UV-B irradiation [14, 15, 16, 17, 18].

The study of biochemical composition of seeds unveils the reason for beneficial effect of *A. aspera* on carps. Two glycosides of oleanolic acid, saponin A and B are present in the alcohol extract of seeds [19]. Chakrabarti et al. [20] reported the occurrence of ecdysterone and two essential fatty acids linolenic acid and oleic acid in the seeds. Ecdysterone is associated with the increased protein synthesis in skeletal muscle [21]. The amino acid profile study shows that leucine, isoleucine, phenylalanine and valine contents of seeds are equivalent to Bengal gram, whereas, sulphur amino acids methionine and cystine contents are higher compared to most pulses [22]. The long-chain polyunsaturated fatty acid (PUFA) plays important role in many physiological functions. So far, all experiments with *A. aspera* are conducted in laboratory conditions. The validation of laboratory study is most essential in the field conditions. Among various parts of the plant, only immunostimulatory properties of roots and seeds have been evaluated; the efficiency of leaves yet to be evaluated.

The innate immune system is the baseline defence system of fish [23]. Myeloperoxidase and nitric oxide synthase reflect the status of the immune system of the species. The elevated levels of these parameters are indicators of healthy immune system of fish. The pattern recognition molecule, the toll-like receptor (TLR) is associated with certain activities of the innate immune system viz. production of cytokines, differentiation of cells, production of reactive nitrogen and oxidative radicals [24, 25]. Cytokines play an important role in the immune system by binding to specific receptors and setting off a cascade of events leading to induction, enhancement or inhibition of a number of cytokines-regulated genes [26]. Interleukin-1β is one of the pivotal early response pro-inflammatory cytokines that enables organisms to respond to any infection, inducing an inflammatory cascade, along with other defensive responses [23, 27]. TNF-α is associated with recruitment and activation of phagocytes [28]. The expression patterns of immune related genes were studied in rohu infected with *Edwardsiella tarda* [29] and *Aeromonas hydrophila* [10, 30, 31]. The information on the expression of immune related genes in fish fed with enriched diets and challenged with bacteria in pond conditions is lacking. The aim of the present study is to evaluate the immunostimulatory and disease resistance properties of seeds and leaves of *Achyranthes aspera* in *Labeo rohita* challenged with *Aeromonas hydrophila* in the pond conditions.

## 2 Materials and methods

### 2.1 Culture of fish and challenge with pathogen

Indian major carp *L. rohita* fry were procured from a fish farm, acclimated for 7 days and then fry (1.9 ± 0.08 g) were introduced in hapas (25 hapa^−1^) set inside the pond (54.5 m × 30.5 m × 2.25 m) of Rohtak Centre, Central Institute of Fisheries Education (Indian Council of Agricultural Research), Haryana. Each hapa (2.0 m × 1.5 m × 1.5 m) was made of nylon net; the top of the hapa was covered with a net to avoid the escape of fish. Hapas were set inside the pond with bamboo stick. Rohu fry were fed with two experimental diets containing plant ingredients and control diet (Table 1). The experimental diets were prepared with 0.5% seeds (diet 1, D1) and leaves (diet 2, D2) of *A. aspera* with other ingredients; diet without plant ingredient served as control (diet 3, D3). *A. aspera* was grown in the outdoor facility; leaves and ripe seeds were collected regularly, cleaned, dried, ground and kept in the refrigerator for further use. Three replicates were used for each feeding regime. Food was given once daily (9.00 a. m.) at the rate of 5% of body weight. Major water quality parameters like temperature, pH, dissolved oxygen and conductivity were recorded regularly using HQ40d Multiparameter (Hach, USA) from four sides of the pond closer to the hapas. Water temperature and pH ranged from 28.5 to 31 °C and 8.4 to 9.16, respectively throughout the culture period. Dissolved oxygen level ranged from 7.11 to 9.62 mg L^−1^ in the pond. After 80 days of initial feeding, fish were anesthetized with MS 222 (Sigma, USA) and injected intraperitoneally (100 μL) with live *Aeromonas hydrophila* (5 × 10^6^ CFU mL^−1^). A group of fish injected with buffer served as sham control. All fish were introduced in the respective hapa and mortality of fish was recorded for 10 days at 12 h interval. The whole study was conducted following the guidelines of Institutional Animal Ethics Committee, IAEC (approved by Committee for the Purpose of Control and Supervision of Experiments on Animals, CPCSEA) (Reference: DU/ZOOL/IAEC-R/2015/08). The study was conducted following all regulations.

**Table 1:** Composition of experimental and control diets.

Ingredients	Diets		
	0.5% Seeds (D1)	0.5% Leaves (D2)	Control (D3)
**Composition (g kg**^**−1**^**)**			
Fish powder	482.76	482.76	482.76
Wheat flour	488.24	488.24	493.24
Cod liver oil	20.00	20.00	20.00
Vitamin & mineral pre-mix	4.00	4.00	4.00
Leaves	-	5.00	-
Seeds	5.00	-	-
**Proximate composition (g 100 g**^**−1**^**)**			
Crude protein	36.71	35.67	33.58
Crude fat	8.31	7.82	8.64
Carbohydrates	37.52	38.36	43.45
Ash	8.03	8.12	7.20
Moisture	9.43	10.03	7.14
Crude fibre	0.44	0.65	0.41
Energy (kcal 100 g^−1^)	371.7	366.5	385.8

### 2.2 Collection of samples

After 10 days of challenge test, fish were anesthetized with MS 222 (Sigma), blood sample was drawn from the caudal vein of each fish using Dispo van 2 mL single use syringe (0.55 × 25 mm/24 × 1) and transferred in serological tube. Blood samples were allowed to clot at 4 °C overnight. The serum was then spun down at 400 × g for 10 min. Then the serum was stored in sterile tube at −20 °C until used for assays. Three replicates were used for each feeding regime. Hepatopancreas and head kidney were collected aseptically and were preserved at −80 °C for biochemical assays and gene expression study.

### 2.3 Myeloperoxidase and nitric oxide synthase assays

Myeloperoxidase activity was measured following the standard method [32]. First 10 μL serum was taken in each well of microplate, then 90 μL of Hank's balanced salt solution (without Ca^2+^ or Mg^2+^), 35 μL of 20 mM 3,3′,5,5′-tetramethylbenzidine hydrochloride (Genei, India) and 5 mM H_2_O_2_ were added to each well of microplate. The mixture was incubated for 2 min and 35 μL of 4 M sulphuric acid was added to stop the reaction. The activity was recorded at 450 nm.

Nitric oxide synthase levels of tissues (hepatopancreas and head kidney) were recorded [33]. In 1 mL phosphate buffer (pH 7.4), 100 mg tissue was homogenized; centrifuged at 10000× g for 20 min at 4 °C. The supernatant, 100 μL was mixed with 100 μL Griess reagent and incubated 10 min at room temperature. The activity was recorded at 540 nm. The nitrite concentration was measured with the nitrite standard curve and expressed as mmol mg tissue^−1^.

### 2.4 Oxidation of tissue lipids and proteins

Thiobarbituric acid reactive substances (TBARS) indicate the oxidation of tissue lipids (hepatopancreas/head kidney). In 9 mL of KCl (1.15%), 1 g tissue was homogenized. Then the sample was incubated at 100 °C for 1 h in acid medium containing 0.45% sodium dodecyl sulphate (SDS) and 0.6% thiobarbituric acid [34]. The sample was centrifuged at 800 x g at 4 °C for 15 min. The standard was prepared with 1, 1, 3, 3-tetramethoxy propane. The activity was measured at 532 nm and expressed as mmol MDA mg protein^−1^.

Carbonyl protein is an indicator of tissue protein oxidation. A 100 mg tissue (hepatopancreas/head kidney) was homogenized in 1 mL of potassium phosphate buffer (50 mM, pH 7.0) containing 0.5 mM ethylenediaminetetraacetic acid and 100 μM of phenylmethlysulfonyl fluoride [35]. The homogenate (250 μL) was mixed with 0.5 ml of 10% TCA, centrifuged at 13000× g for 5 min and the pellet was used for the assay. The pellet was mixed with 10 mM dinitrophenyl hydrazine (1 mL, DNPH) dissolved in 2 M HCl. It was incubated 1 h at room temperature, centrifuged at 13000 x g for 5 min; the pellet was collected and washed thrice with 1 mL of ethanol-butylacetate (1:1, v/v). Then it was dissolved in 1.5 mL of 6 M guanidine hydrochloride, centrifuged at 13000× g for 5 min and the supernatant was collected. The optical density of the supernatant was measured at 370 nm. The result was expressed as nmol mg protein^−1^. The molar extinction coefficient was 22 × 10^3^ M^−1^ cm^−1^. Total tissue protein was measured at 750 nm [36].

### 2.5 Cumulative mortality rate of fish

The mortality of rohu in each hapa was recorded at 12 h interval after the challenge with *A. hydrophila*. The number of fish was recorded and expressed in per cent with respect to the initial one. Finally, it was expressed as cumulative mortality rate of rohu.

### 2.6 Gene expression study

#### 2.6.1 Total RNA isolation and cDNA synthesis

The hepatopancreas and head kidney (100 mg) of rohu were processed separately in TRIzol reagent (Ambion, Life Technologies, USA) for the extraction of total RNA following the protocol of manufacturer. In Synergy H1 Hybrid microplate reader (Biotek, USA), the concentration and purity of RNA was checked at 260 and 280 nm, using Take 3 plate. Then integrity of extracted RNA was checked in 1% agarose gel. Total RNA (1 μg), was treated with 1U of DNase I (Sigma-Aldrich, USA) to avoid any contamination of DNA. High capacity cDNA reverse transcription kit (Applied Biosystems, USA) with RNase inhibitor was used for the reverse transcription of DNase-treated RNA following the manufacturer's protocol. The product was stored at −20 °C for further gene expression study.

#### 2.6.2 Quantitative real-time PCR analysis

The quantitative real-time PCR (qRT-PCR) was performed to amplify immune relevant target genes like, lysozyme G, lysozyme C, TNF-α, IL-10, IL-1β, TLR-4 and housekeeping gene, β-actin using QuantStudio 6 Flex Real-Time PCR system (Applied Biosystems). MicroAmp optical 96-well reaction plate (0.1 mL) was used for amplifications. Primers for different genes were either self-designed or collected from earlier published data (Table 2). The cDNA of all the treatments were diluted (1:1) in nuclease-free water. Amplifications were carried out with 10 μL reaction volume and that composed of 1.0 μL of diluted cDNA, 0.25 μL of Forward and Reverse primers (2.5 μM each), 5 μL of Universal 2X PowerUp SYBR Green Master Mix (Applied Biosystems), 3.5 μL of nuclease-free water. Amplifications were performed in duplicate wells under following conditions: initial denaturation at 95 °C for 10 min followed by 40 cycles of 95 °C for 15 sec, annealing at 55 °C (for lysozyme G, lysozyme C, TNF-α) and 60 °C (for IL-10, IL-1β, TLR-4, β-actin), and extension at 72 °C for 1 min. The reaction carried out without cDNA template was a negative control. The qPCR specificity was verified with melt curve analysed at a 95 °C for 15 sec (1.6 °C sec^−1^), 60 °C for 1 min (1.6 °C sec^−1^) and 95 °C for 15 sec (0.15 °C sec^−1^). The PCR efficiencies were determined by analysis of serial dilutions of cDNA. The efficiencies close to 100% allowed the application of 2^−ΔΔCT^ method for calculation of relative gene expression of the target gene with that of reference gene β-actin [37]. The specificity of the amplification product was verified in 1% agarose gel.

**Table 2:** Target genes and sequences of primers used for qPCR analysis.

Target gene	Primer	Primer sequence (5′-3′)	Accession number/reference
Lysozyme C	Lyso C Fw Lyso C Rv	CGATGATGGCACTCCAGGT CATGCTTTCAGTCCTTCGGC	EF203085.1
Lysozyme G	Lyso G Fw Lyso G Rv	CAATGGCTTTGGCCTCATGC CACGTGGGAAACTTTGCTCTG	KC934746.1
TNF-α	TNF-α Fw TNF-α Rv	GGCGGCTTGAAAGTAGTGGA TATGCAGAACGTCGTGGTCC	FN543477.1
IL-10	IL-10 Fw IL-10 Rv	GCTCAGTGCAGAAGAGTCGAC CCCGCTTGAGATCCTGAAATATA	[49]
IL-1β	IL-1β Fw IL-1β Rv	GTACCCCACAAAACATCGGC CAAGAGCAGTTTGGGCAAGG	AM932525.1
TLR-4	TLR-4 Fw TLR-4 Rv	CTAAGAAAGTGCTTGGGCTTCAT GGTTTGTGGCAATAATGGCTTTC	KX218428.1
β-actin	β-actin Fw β-actin Rv	GACTTCGAGCAGGAGATGG CAAGAAGGATGGCTGGAACA	[29]

### 2.7 Statistical analysis

All data are given as Mean ± SE. One-way analysis of variance and Duncan's multiple range test [38] were used to find out the difference among various treatments. Statistical significance was accepted at *P* < 0.05 level.

## 3 Results

### 3.1 Cumulative mortality of rohu

**Fig. 1:**
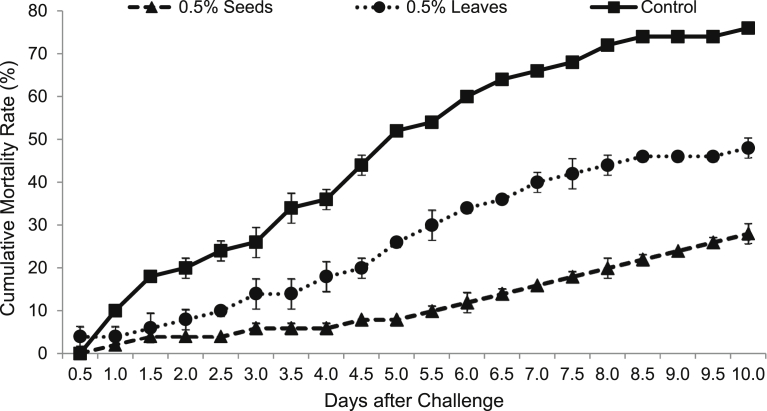
Cumulative mortality of *L. rohita* fed with three different diets and challenged with *A. Hydrophila*.

Rohu fry were fed with test diets containing 0.5% seeds (D1) and leaves (D2) of *A. aspera* and control diet (D3) for initial 80 days and then challenged with *A. hydrophila*. After bacterial challenge, the mortality of fish was recorded for 10 days at 12 h interval. First mortality was recorded in D2 diet fed rohu within 12 h of challenge with bacterial pathogen. The mortality rates were 2, 4 and 10% in D1, D2 and D3 diets fed groups, respectively after 24 h of challenge. In control diet fed group, 50% fish died within five days of challenge. The cumulative mortality rate was significantly (*P* < 0.05) higher in control diet fed rohu compared to the seeds and leaves supplemented diets fed fish (Fig. 1). The cumulative mortality rates were 28, 48 and 76% in D1, D2 and D3 diets fed rohu, respectively.

### 3.2 Average weight

Plant supplemented diet showed a positive impact on the growth of rohu fry. The average weight was significantly (*P* < 0.05) higher in rohu fed with 0.5% seeds supplemented diet (D1) compared to other two feeding regimes. The average weight of D1 diet fed rohu was 6.5 and 12.5% higher compared to the D2 and D3 diets fed fish, respectively (Table 3).

**Table 3:** Initial and final average weights of *L. rohita* fed with three different diets and challenged with *A. hydrophila*. Mean ± SE (25 fish hapa^−1^, 3 hapas treatment^−1^; 25 × 3 = 75 in each treatment) sharing different letters in the same column are significantly (*P* < 0.05) different.

Parameters	(D1) 0.5% Seeds	(D2) 0.5% Leaves	(D3) Control
Initial weight (g)	1.9 ± 0.08a	1.9 ± 0.08a	1.9 ± 0.08a
Average weight (g)	25 ± 0.40a	23.50 ± 0.22b	22.22 ± 0.11c

### 3.3 Myeloperoxidase and nitric oxide synthase assays

Myeloperoxidase activity was significantly (*P* < 0.05) higher in D1 and D2 diets fed rohu compared to the control diet fed fish. There was no significant (*P* > 0.05) difference in myeloperoxidase activity between the former two groups. Nitric oxide synthase levels in hepatopancreas and kidney were significantly (*P* < 0.05) higher in D1 diet fed rohu compared to others. The level was 5–7 folds higher in hepatopancreas compared to the kidney of rohu fed with same diet (Table 4).

**Table 4:** Myeloperoxidase, nitric oxide synthase, thiobarbituric acid reactive substances (TBARS) and carbonyl protein levels found in *L. rohita* fed with three different diets and challenged with *A. hydrophila*. Mean ± SE (6 fish hapa^−1^, 3 hapas treatment^−1^; 6 × 3 = 18 in each treatment) sharing different letters in the same column are significantly (*P* < 0.05) different.

Parameters	0.5% Seeds (D1)	0.5% Leaves (D2)	Control (D3)
**Serum**			
Myeloperoxidase (O.D. at 450 nm)	0.92 ± 0.04a	0.91 ± 0.02a	0.62 ± 0.03b
**Hepatopancreas**			
Nitric oxide synthase (μmol mg tissue^−1^)	20.82 ± 0.14a	15.25 ± 0.26b	15.36 ± 0.20b
TBARS (mmol MDA mg protein^−1^)	0.98 ± 0.02c	1.32 ± 0.04b	2.94 ± 0.03a
Carbonyl protein (nmol mg protein^−1^)	7.80 ± 0.04c	8.56 ± 3.16b	16.71 ± 5.05a
**Head kidney**			
Nitric oxide synthase (μmol mg tissue^−1^)	4.30 ± 0.02a	2.74 ± 0.02b	1.78 ± 0.02c
TBARS (mmol MDA mg protein^−1^)	0.75 ± 0.01c	1.97 ± 0.05b	3.25 ± 0.05a
Carbonyl protein (nmol mg protein^−1^)	5.48 ± 0.19c	7.78 ± 0.27b	17.99 ± 0.28a

### 3.4 Oxidation of tissue lipids and proteins

TBARS levels were significantly (*P* < 0.05) lower in hepatopancreas and kidney of D1 diet fed rohu compared to others. Similarly, carbonyl protein level was significantly (*P* < 0.05) lower in D1 diet fed rohu compared to others. Highest TBARS and carbonyl protein levels were found in D3 diet fed rohu. The levels were higher in hepatopancreas compared to the kidney of rohu cultured in the same feeding regime (Table 4).

### 3.5 Gene expression

**Fig. 2:**
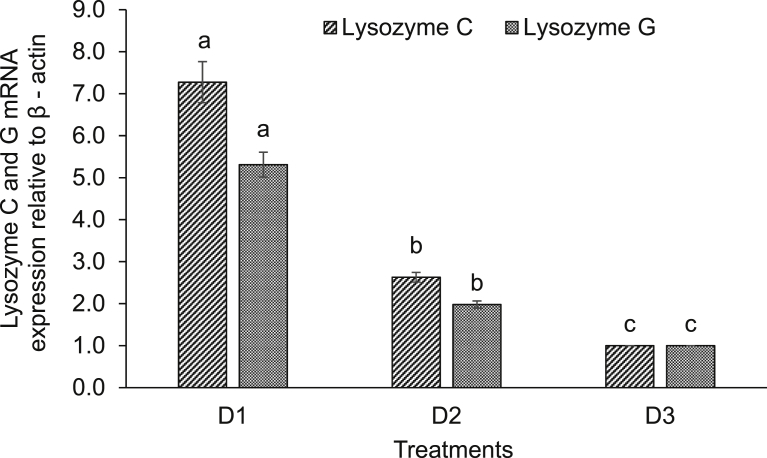
Expression of lysozyme C and lysozyme G in hepatopancreas of *L. rohita* fed with three different diets and challenged with *A. hydrophila*. The relative expression of the target gene lysozyme C/G was normalized to the expression of β-actin (internal control) and expressed as fold changes relative to the control diet fed group. Vertical bars with different superscripts are significantly (*P* < 0.05) different (2 fish hapa^−1^, 3 hapas treatment^−1^; 2 × 3 = 6).

**Fig. 3:**
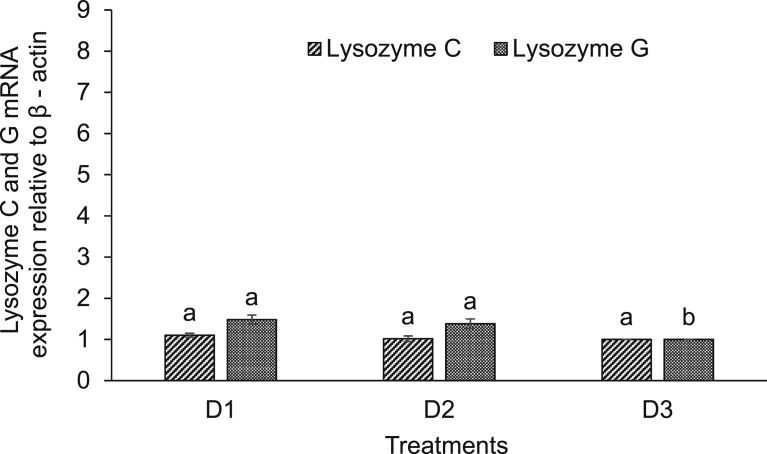
Expression of lysozyme C and lysozyme G in kidney of *L. rohita* fed with three different diets and challenged with *A. hydrophila*. The relative expression of the target gene lysozyme C/G was normalized to the expression of β-actin (internal control) and expressed as fold changes relative to the control diet fed group. Vertical bars with different superscripts are significantly (*P* < 0.05) different (2 fish hapa^−1^, 3 hapas treatment^−1^; 2 × 3 = 6).

**Fig. 4:**
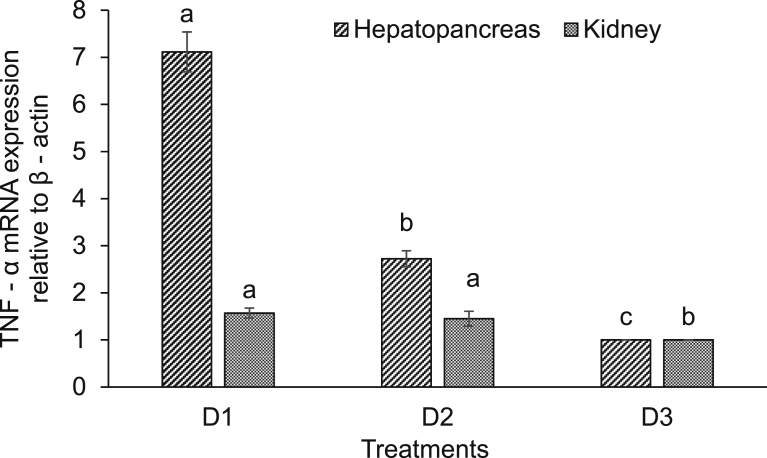
Expression of TNF-α in hepatopancreas and kidney of *L. rohita* fed with three different diets and challenged with *A. hydrophila*. The relative expression of the target gene TNF-α was normalized to the expression of β-actin (internal control) and expressed as fold changes relative to the control diet fed group. Vertical bars with different superscripts are significantly (*P* < 0.05) different (2 fish hapa^−1^, 3 hapas treatment^−1^; 2 × 3 = 6).

**Fig. 5:**
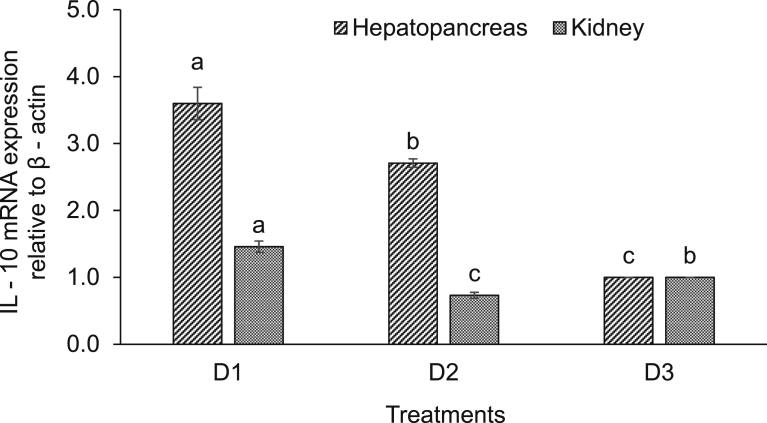
Expression of IL-10 in hepatopancreas and kidney of *L. rohita* fed with three different diets and challenged with *A. hydrophila*. The relative expression of the target gene IL-10 was normalized to the expression of β-actin (internal control) and expressed as fold changes relative to the control diet fed group. Vertical bars with different superscripts are significantly (*P* < 0.05) different (2 fish hapa^−1^, 3 hapas treatment^−1^; 2 × 3 = 6).

**Fig. 6:**
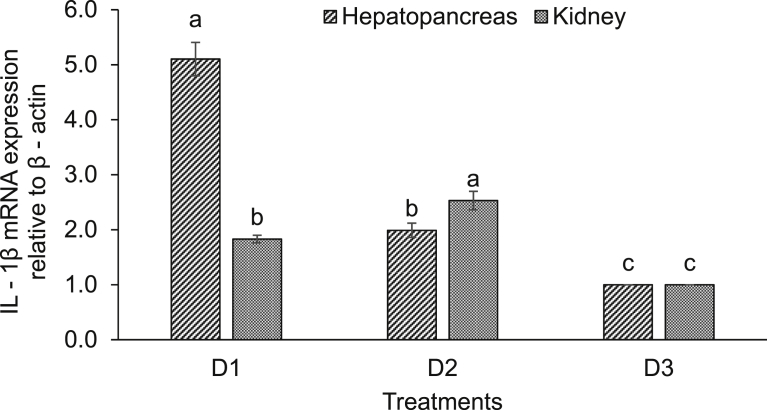
Expression of IL-1β in hepatopancreas and kidney of *L. rohita* fed with three different diets and challenged with *A. hydrophila*. The relative expression of the target gene IL-1β was normalized to the expression of β-actin (internal control) and expressed as fold changes relative to the control diet fed group. Vertical bars with different superscripts are significantly (*P* < 0.05) different (2 fish hapa^−1^, 3 hapas treatment^−1^; 2 × 3 = 6).

**Fig. 7:**
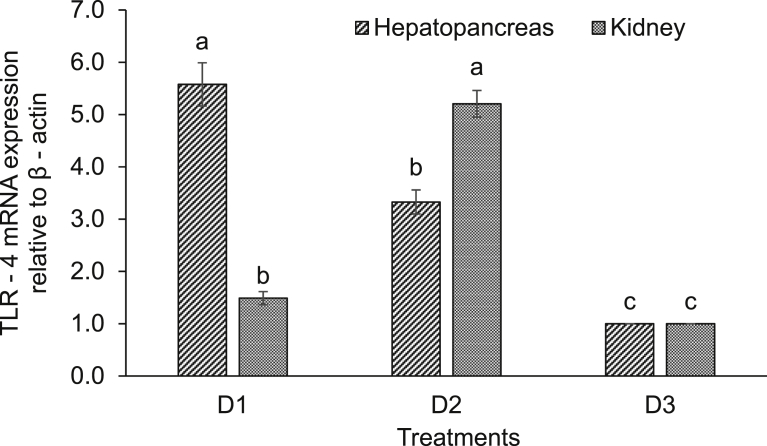
Expression of TLR-4 in hepatopancreas and kidney of *L. rohita* fed with three different diets and challenged with *A. hydrophila*. The relative expression of the target gene TLR-4 was normalized to the expression of β-actin (internal control) and expressed as fold changes relative to the control diet fed group. Vertical bars with different superscripts are significantly (*P* < 0.05) different (2 fish hapa^−1^, 3 hapas treatment^−1^; 2 × 3 = 6).

Expression of various immune-related genes supported the physiological study. In hepatopancreas of seeds (D1) supplemented diet fed rohu, the expressions of lysozyme C and G were significantly (*P* < 0.05) higher compared to others. This treatment was followed by D2 diet fed rohu and minimum level was found in control diet (D3) fed fish (Fig. 2). The expression of lysozyme C was higher compared to lysozyme G in the hepatopancreas of rohu cultured in the same feeding regime. Lysozyme G levels were significantly (*P* < 0.05) higher in kidney of rohu fed with D1 and D2 diets compared to the D3 diet fed fish (Fig. 3). There was no change in lysozyme C level in plant supplemented diets fed rohu compared to the control diet fed fish. In hepatopancreas of D1 diet fed rohu, the expression of TNF-α was significantly (*P* < 0.05) higher compared to others. This treatment was followed by D2 and D3 diets fed rohu (Fig. 4). In kidney, the expression of TNF-α was significantly (*P* < 0.05) higher in D1 and D2 diets fed rohu compared to D3. The expression of TNF-α was 2–6 folds higher in hepatopancreas compared to kidney of rohu cultured in same feeding regime. There was up-regulation (*P* < 0.05) of IL-10 in hepatopancreas of rohu fed with D1 diet compared to others (Fig. 5). This treatment was followed by D2 diet fed rohu. In kidney, IL-10 was up-regulated (*P* < 0.05) only in D1 diet fed rohu and it was down-regulated in D2 diet fed fish compared to the control diet fed fish. In hepatopancreas of D1 diet fed rohu, IL-1β was up-regulated (*P* < 0.05) compared to others (Fig. 6). Whereas, in kidney significantly (*P* < 0.05) higher expression was found in D2 diet fed fish compared to others. Similar trend was also found with TLR-4. There was up-regulation (*P* < 0.05) of TLR-4 in hepatopancreas and kidney of rohu fed with D1 and D2 diets, respectively compared to D3 diet fed fish (Fig. 7).

## 4 Discussion

The feeding of rohu fry with *A. aspera* seeds and leaves enriched diets showed very positive effect even in the pond conditions. The mortality rate was highest in the control diet fed fish. The cumulative mortality rate of fish was 28–48% reduced in enriched diet fed rohu. Like earlier laboratory based study, best result was obtained in the seeds supplemented diets fed rohu compared to the other feeding regimes. This is the first report showing immunostimulatory and disease resistance properties of leaves of *A. aspera*. In laboratory experiments, early larvae and fingerlings of rohu were fed with seeds supplemented diets and then challenged with *A. hydrophila*. Significantly lower mortality was found in seeds supplemented diets fed rohu compared to the control one [10, 30]. This is clear from the study that *A. aspera* provides protection against bacterial pathogen to all age groups of rohu in control laboratory conditions as well as in natural pond.

Like earlier study conducted in the laboratory conditions, growth stimulatory property of plant supplemented diet was observed in rohu fry cultured in pond. The average weight was 6.5–12.5% higher in rohu fed with 0.5% seeds supplemented diet compared to other two feeding regimes in the present study. Enhanced growth of fish was also found in leaves supplemented diet fed fish compared to the control one in the pond study. Giri et al. [6] found that supplementation of leaves of other plant, the guava leaves at 0.5% level enhanced the growth of rohu compared to the control diet fed fish. The growth of fish is an indicator of health status that influences the other physiological conditions. In a laboratory study, common carp larvae fed with 0.5% seed supplemented diet showed higher average weight compared to the fish fed with control diet [20]. Enhanced growth and survival are the basic requirements of successful aquaculture. In the present study, these two primary requisites are achieved in the pond conditions. The study of immunological and oxidative stress parameters and gene expression supported these two basic observations.

Higher levels of myeloperoxidase and nitric oxide synthase showed the efficient immune system of rohu in the present study. This pond study confirmed the earlier laboratory experiments. Enrichment of diets with plant ingredients improved the efficiency of the immune system. Seeds are superior to leaves. A tissue-specific physiological activity was recorded in the present study as nitric oxide synthase level was always higher in hepatopancreas compared to the kidney of rohu cultured in the same feeding regime. Myeloperoxidase and nitric oxide synthase are indicators of the immunological status of the fish. Myeloperoxidase shows antimicrobial activity. It occurs abundantly in neutrophil granulocytes; elevated level of myeloperoxidase helps in the destruction and elimination of invading pathogens from the host body [39, 40]. Nitric oxide synthase catalyzes the production of cellular signalling molecule nitric oxide that plays vital role in defence mechanism of fish [41]. In the present study, the elevated levels of myeloperoxidase and nitric oxide synthase indicated the improved defence system of rohu fed with plant supplemented diets. Supplementation of seeds of *A. aspera* enhanced the nitric oxide synthase level in catla [42]. The presence of long-chain polyunsaturated fatty acids such as linolenic and oleic acids in the seeds [20] may be associated with the immunostimulatory properties of rohu challenged with bacterial pathogen in the present study.

Feeding of fish with enriched diets stimulated the immune system and as well as reduced the stress in fish [14, 24, 26]. This was evident from the lower levels of TBARS and carbonyl protein in hepatopancreas and kidney of enriched diets fed fish compared to the control fish in the present study. The study also showed that hepatopancreas was more sensitive to stress compared to kidney as TBARS and carbonyl protein levels were highest in the former regardless of feeding regime. Seeds showed better performance in stress reduction compared to the leaves; still the latter was better compared to the control treatment. Elevated levels of TBARS and carbonyl protein are indicators of oxidation of tissue lipid and protein, respectively. Lipid peroxidation is a well-established mechanism of oxidative damage caused by reactive oxygen species [43]. Lipid peroxidation is the process of oxidative degradation of PUFA and its occurrence in biological membranes causes impaired membrane function, structural integrity and inactivation of several membrane-bound enzymes [44]. Lipid peroxidation may bring about protein damage by its end products, MDA and 4-hydroxynonenal [45]. Fish challenged with bacterial pathogen were prone to oxidation of lipid and protein. Dietary supplementation of plant ingredients reduced oxidative stress in rohu in the present study.

In the present study, lysozyme C, lysozyme G, TNF-α, IL-10, IL-1β and TLR-4 were up-regulated in hepatopancreas of enriched diets fed rohu compared to the control one after challenged with *A. hydrophila*. The expressions were higher in seeds supplemented diet fed rohu compared to the fish fed with leaves supplemented diet. In kidney of same rohu, most of these genes were up-regulated compared to the control diet fed fish, except lysozyme C and IL-10. IL-10 was down-regulated in leaves supplemented diet fed rohu compared to the control diet fed fish. In kidney, lysozyme G, TNF-α, IL-10 expressions were higher in the seeds supplemented diet fed rohu compared to the leaves supplemented diet fed fish, whereas, expressions of IL-1β and TLR-4 showed the opposite trend. The feeding of rohu fingerlings with other plant ingredient, ginger (root of the plant) showed up-regulation of IL-10, transforming growth factor-beta (TGF-β) in head kidney, intestine and hepatopancreas [8]. A significant difference was found in the expression pattern of IL-1β, TNF-α, lysozyme C and lysozyme G in *E. tarda* infected and un-infected rohu [29]. In rohu challenged with *A. hydrophila*, a significant up-regulation of IL-10 and down-regulation of IL-1β and TNF-α was reported in treated group compared to control [30]. This showed the response of these genes in presence of pathogen. In rohu fed with other plant leaves, the guava leaves supplemented diets, there were up-regulations of IL-1β, TNF-α in head-kidney, intestine and hepatopancreas, whereas, IL-10 was down-regulated [6]. Significantly higher expressions of lysozyme, TNF-α and IL-1β were observed in common carp fed with jujube *Ziziphus jujube* [1], ferula *Ferula assafoetida* [46] and loquat *Eriobotrya japonica* [47]. In another study, common carp fed with guava leaf powder enriched diets showed significant up-regulation of IL-1β while there was no change in the expression of TNF-α [7]. The expressions of NOD 1 and TLR-22 were higher in liver compared to kidney [48]. Tissue-specific expressions of various genes were reported in catla [42]. Similar results were also found in the pond experiment with rohu as the expressions of various genes were higher in hepatopancreas compared to kidney of fish cultured in the same feeding regime.

## 5 Conclusions

In conclusions, dietary supplementation of *A. aspera* seeds and leaves enhanced the growth of rohu, induced the immune system, reduced oxidative damage of tissue and protected fish from bacterial pathogen. There were up-regulations of most of the genes in enriched diets fed rohu compared to the control group. Seeds showed better performance compared to the leaves. The bioactive compounds present in seeds are beneficial for the fish health. This plant ingredient has immense prospect in the production of healthy fish in ponds.

## Declarations

### Author contribution statement

Rina Chakrabarti: Conceived and designed the experiments; Performed the experiments; Analyzed and interpreted the data; Contributed reagents, materials, analysis tools or data; Wrote the paper.

Neelesh Kumar: Performed the experiments; Analyzed and interpreted the data; Wrote the paper.

Jaigopal Sharma: Conceived and designed the experiments; Performed the experiments; Contributed reagents, materials, analysis tools or data; Wrote the paper.

Samar Pal Singh: Performed the experiments; Analyzed and interpreted the data.

Amarjeet Singh, V Harikrishna: Performed the experiments.

### Funding statement

This work was supported by the Department of Biotechnology, DBT, Government of India, New Delhi (102/IFD/SAN/4260/2014–2015).

### Competing interest statement

The authors declare no conflict of interest.

### Additional information

No additional information is available for this paper.

## References

[1] Hoseinifar S.H., Zou H.K., Paknejad H., Ahmadifar E., Doan H.V. (2018). Non-specific immune responses and intestinal immunity of common carp (*Cyprinus carpio*) fed jujube (*Ziziphus jujube*) fruit extract. Aquacult. Res..

[2] Sudhakaran D.S., Srirekha P., Devasree L.D., Premsingh S., Michael R.D. (2006). Immunostimulatory effect of *Tinospora cordifolia* miers leaf extract in *Oreochromis mossambicus*. Indian J. Exp. Biol..

[3] Sahu S., Das B.K., Pradhan J., Mohapatra B.C., Mishra B.K., Sarangi N. (2007). Effect of *Magnifera indica* kernel as a feed additive on immunity and resistance to *Aeromonas hydrophila* in *Labeo rohita* fingerlings. Fish Shellfish Immunol..

[4] Divyagnaneswari M., Christybapita D., Michael R.D., Bondad-Reantaso M.G., Mohan C.V., Crumlish M., Subasinghe R.P. (2008). Immunomodulatory activity of *Solanum trilobatum* leaf extracts in *Oreochromis mossambicus*. Diseases in Asian Aquaculture VI, Fish Health Section.

[5] Harikrishnan R., Balasundaram C., Heo M.S. (2011). Impact of plant products on innate and adaptive immune system of cultured finfish and shellfish. Aquaculture.

[6] Giri S.S., Sen S.S., Chi C., Kim H.J., Yun S., Park S.C., Sukumaran V. (2015). Effect of guava leaves on the growth performance and cytokine gene expression of *Labeo rohita* and its susceptibility to *Aeromonas hydrophila* infection. Fish Shellfish Immunol..

[7] Hoseinifar S.H., Sohrabi A., Paknejad H., Jafari V., Paolucci M., Doan H.V. (2018). Enrichment of common carp (*Cyprinus carpio*) fingerlings diet with *Psidium guajava*: the effects on cutaneous mucosal and serum immune parameters and immune related genes expression. Fish Shellfish Immunol..

[8] Sukumaran V., Park S.C., Giri S.S. (2016). Role of dietary ginger *Zingiber officinale* in improving growth performances and immune functions of *Labeo rohita* fingerlings. Fish Shellfish Immunol..

[9] Rao V.Y., Romesh M., Singh A., Chakrabarti R. (2004). Potentiation of antibody production in Indian major carp *Labeo rohita*, rohu by *Achyranthes aspera* as an herbal feed ingredient. Aquaculture.

[10] Rao Y.V., Das B.K., Jyotyrmayee P., Chakrabarti R. (2006). Effect of *Achyranthes aspera* on the immunity and survival of *Labeo rohita* infected with *Aeromonas hydrophila*. Fish Shellfish Immunol..

[11] Rao Y.V., Chakrabarti R. (2005). Stimulation of immunity in Indian major carp *Catla catla* with herbal feed ingredients. Fish Shellfish Immunol..

[12] Chakrabarti R., Rao Y.V. (2006). *Achyranthes aspera* stimulate the immunity and enhances the antigen clearance in *Catla catla*. Int. Immunopharmacol..

[13] Chakrabarti R., Rao Y.V. (2012). *Achyranthes aspera* enhances the immunity and antigen clearance in common carp *Cyprinus carpio*. J. Fish. Dis..

[14] Singh K.M., Sharma J.G., Chakrabarti R. (2013). Impact of UV-B radiation on the physiology of freshwater carp *Labeo rohita* larvae and evaluation of UV-B protective properties of seeds of *Achyranthes aspera* and vitamin C. Agric. Res..

[15] Singh K.M., Sharma J.G., Chakrabarti R. (2013). Effect of UV-B radiation on the defence system of *Labeo rohita* (Actinopterygii: Cypriniformes: Cyprinidae) larvae and its modulation by seed of Devil's Horsewhip *Achyranthes aspera*. Acta Ichthyol. Piscat..

[16] Singh K.M., Sharma J.G., Chakrabarti R. (2015). Simulation study of natural UV-B radiation on *Catla catla* and its impact on physiology, oxidative stress, Hsp70 and DNA fragmentation. Photochem. Photobiol..

[17] Sharma J.G., Rao Y.V., Kumar S., Chakrabarti R. (2010). Impact of UV-B radiation on the digestive enzymes and immune system of larvae of Indian major carp *Catla catla*. Int. J. Radiat. Biol..

[18] Sharma J.G., Singh M.K., Chakrabarti R. (2015). Physiological responses of *Catla catla* larvae fed with *Achyranthes aspera* seed enriched diet and exposed to UV-B radiation. Indian J. Biochem. Biophys..

[19] Hariharan V., Rangaswami S. (1970). Structure of saponins A and B from the seeds of *Achyranthes aspera*. Phytochemistry.

[20] Chakrabarti R., Srivastava P.K., Kundu K., Khare R.S., Banerjee S. (2012). Evaluation of immunostimulatory and growth promoting effect of seed fractions of *Achyranthes aspera* in common carp *Cyprinus carpio* and identification of active constituents. Fish Shellfish Immunol..

[21] Gorelick-Feldman J., Maclean D., Ilic N., Poulev A., Lila M.A., Cheng D., Raskin I. (2008). Phytoecdysteroids increase protein synthesis in skeletal muscle cells. J. Agric. Food Chem..

[22] Goyal B.R., Goyal R.K., Mehta A.A. (2007). Phyto-pharmacology of *Achyranthes aspera*: a review. Pharmacogn. Rev..

[23] Belvin M.P., Anderson K.V. (1996). A conserved signaling: the *Drosophila* Toll-dorsal pathway. Annu. Rev. Cell Dev. Biol..

[24] Aoki T., Hirono I. (2006). Immune relevant genes of Japanese flounder, *Paralichthys olivaceus*. Comp. Biochem. Physiol..

[25] Lee T.I., Rinaldi N.J., Robert F., Odom D.T., Joseph Z.B., Gerber G.K., Hannett N.M., Harbison C.T., Thompson C.M., Simon I., Zeitlinger J., Jennings E.G., Murray H.L., Gordon D.B., Ren B., Wyrick J.J., Tagne J.B., Volkert T.L., Fraenkel E., Gifford D.K., Young R.A. (2002). Transcriptional regulatory networks in *Saccharomyces cerevisiae*. Science.

[26] Ellis A.E. (2001). Innate host defense mechanisms of fish against viruses and bacteria. Dev. Comp. Immunol..

[27] Magnadottir B. (2006). Innate immunity of fish (overview). Fish Shellfish Immunol..

[28] Roca F.J., Mulero I., Munoz A.L., Sepulcre M.P., Renshaw S.A., Meseguer J., Mulero V. (2008). Evolution of the inflammatory response in vertebrates: fish TNF-α is a powerful activator of endothelial cells but hardly activates phagocytes. J. Immunol..

[29] Mohanty B.R., Sahoo P.K. (2010). Immune responses and expression profiles of some immune-related genes in Indian major carp, *Labeo rohita* to *Edwardsiella tarda* infection. Fish Shellfish Immunol..

[30] Swain B., Basu M., Samanta M. (2011). Cloning of interleukin-10 gene in the Indian major carp, *Labeo rohita* (Hamilton 1822) and its functional characterization following *Aeromonas hydrophila* infection. Indian J. Fish..

[31] Chakrabarti R., Srivastava P.K. (2012). Effect of dietary supplementation of *Achyranthes aspera* seed on *Labeo rohita* larvae challenged with *Aeromonas hydrophila*. J. Aquat. Anim. Health.

[32] Quade M.J., Roth J.A. (1997). A rapid, direct assay to measure degranulation of bovine neutrophil primary granules. Vet. Immunol. Immunopathol..

[33] Lee D.U., Kang Y.J., Park M.K., Lee Y.S., Seo H.G., Kim T.S., Kim C.H., Chang K.C. (2003). Effects of 13-alkyl-substituted berberine alkaloids on the expression of COX-II, TNF-alpha, iNOS, and IL-12 production in LPS-stimulated macrophages. Life Sci..

[34] Ohkawa H., Ohishi N., Yagi K. (1979). Assay for lipid peroxides in animal tissues by thiobarbituric acid reaction. Anal. Biochem..

[35] Lenz A.G., Costabel U., Shaltiel S., Levine R.L. (1989). Determination of carbonyl groups in oxidatively modified proteins by reduction with tritiated sodium borohydride. Anal. Biochem..

[36] Lowry O.H., Rosebrough N.J., Farr A.L., Randall R.J. (1951). Protein measurement with the Folin phenol reagent. J. Biol. Chem..

[37] Livak K.J., Schmittgen T.D. (2001). Analysis of relative gene expression data using real-time quantitative PCR and the 2^-ΔΔCT^ method. Methods.

[38] Montgomery D.C. (1984). Design and Analysis of Experiments.

[39] Yano T., Stolen J.S., Fletcher T.C., Anderson D.P., Kaattari S.L., Rowley A.F. (1992). Assays of hemolytic complement activity. Techniques in Fish Immunology.

[40] Dalmo R.A., Ingebrigtsen K., Bøgwald J. (1997). Non-specific defence mechanisms in fish, with particular reference to the reticuloendothelial system (RES). J. Fish. Dis..

[41] Rombout J.H.W.M., Huttenhuis H.B.T., Picchietti S., Scapigliati G. (2005). Phylogeny and ontogeny of fish leucocytes. Fish Shellfish Immunol..

[42] Chakrabarti R., Srivastava P.K., Verma N., Sharma J.G. (2014). Effect of seeds of *Achyranthes aspera* on the immune responses and expression of some immune-related genes in carp *Catla catla*. Fish Shellfish Immunol..

[43] Devasena T., Lalitha S., Padma K. (2001). Lipid peroxidation, osmotic fragility and antioxidant status in children with acute post-streptococcal glomerulonephritis. Clin. Chim. Acta.

[44] Goel A., Dani V., Dhawan D.K. (2005). Protective effects of zinc on lipid peroxidation, antioxidant enzymes and hepatic histoarchitecture in chlorpyrifos-induced toxicity. Chem. Biol. Interact..

[45] Bhor V.M., Raghuram N., Sivakami S. (2004). Oxidative damage and altered antioxidant enzyme activities in the small intestine of streptozotocin-induced diabetic rats. Int. J. Biochem. Cell Biol..

[46] Safari R., Hoseinifar S.H., Nejadmoghadam S., Jafar A. (2016). Transciptomic study of mucosal immune, antioxidant and growth related genes and non-specific immune response of common carp (*Cyprinus carpio*) fed dietary ferula (*Ferula assafoetida*). Fish Shellfish Immunol..

[47] Hoseinifar S.H., Zou H.K., Doan H.V., Miandare H.K., Hoseini S.M. (2017). Evaluation of some intestinal cytokines genes expression and serum innate immune parameters in common carp (*Cyprinus carpio*) fed dietary loquat (*Eriobotrya japonica*) leaf extract. Aquacult. Res..

[48] Kole S., Anand D., Sharma R., Tripathi G., Makesh M., Rajendran K.V., Kadam Bedekar M. (2017). Tissue specific expression profile of some immune related genes in *Labeo rohita* to *Edwardsiella tarda* infection. Fish Shellfish Immunol..

[49] Banerjee S., Mitra T., Purohit G.K., Mohanty S., Mohanty B.P. (2015). Immunomodulatory effect of arsenic on cytokine and HSP gene expression in *Labeo rohita* fingerlings. Fish Shellfish Immunol..

